# Monocytic MKP-1 is a Sensor of the Metabolic Environment and Regulates Function and Phenotypic Fate of Monocyte-Derived Macrophages in Atherosclerosis

**DOI:** 10.1038/srep34223

**Published:** 2016-09-27

**Authors:** Hong Seok Kim, Sina Tavakoli, Leigh Ann Piefer, Huynh Nga Nguyen, Reto Asmis

**Affiliations:** 1 Department of Molecular Medicine, College of Medicine, Inha University, Incheon 22212, Republic of Korea; 2 Hypoxia-related Disease Research Center, College of Medicine, Inha University, Incheon 22212, Republic of Korea; 3 Department of Radiology, University of Texas Health Science Center at San Antonio, San Antonio, TX 78229, USA; 4 Department of Clinical Laboratory Sciences, University of Texas Health Science Center at San Antonio, San Antonio, TX 78229, USA; 5 Department of Biochemistry, University of Texas Health Science Center at San Antonio, San Antonio, TX 78229, USA.

## Abstract

Diabetes promotes the *S*-glutathionylation, inactivation and subsequent degradation of mitogen-activated protein kinase phosphatase 1 (MKP-1) in blood monocytes, and hematopoietic MKP-1-deficiency in atherosclerosis-prone mice accelerates atherosclerotic lesion formation, but the underlying mechanisms were not known. Our aim was to determine the mechanisms through which MKP-1 deficiency in monocytes and macrophages promotes atherogenesis. Transplantation of MKP-1-deficient bone marrow into LDL-R^−/−^ (MKP-1_LeuKO_) mice accelerated high-fat diet (HFD)-induced atherosclerotic lesion formation. After 12 weeks of HFD feeding, MKP-1_LeuKO_ mice showed increased lesion size in both the aortic root (1.2-fold) and the aorta (1.6-fold), despite reduced plasma cholesterol levels. Macrophage content was increased in lesions of MKP-1_LeuKO_ mice compared to mice that received wildtype bone marrow. After only 6 weeks on a HFD, *in vivo* chemotactic activity of monocytes was already significantly increased in MKP-1_LeuKO_ mice. MKP-1 deficiency in monocytes and macrophages promotes and accelerates atherosclerotic lesion formation by hyper-sensitizing monocytes to chemokine-induced recruitment, predisposing macrophages to M1 polarization, decreased autophagy and oxysterol-induced cell death whereas overexpression of MKP-1 protects macrophages against metabolic stress-induced dysfunction. MKP-1 serves as a master-regulator of macrophage phenotype and function and its dysregulation by metabolic stress may be a major contributor to atherogenesis and the progression of atherosclerotic plaques.

Atherosclerosis is a chronic inflammatory disease characterized by monocyte infiltration and macrophage accumulation in the vessel wall^1^. Inflammatory processes are tightly regulated, and macrophages play a critical role in the initiation, maintenance, and resolution of inflammation by converting into distinct activation states, ranging from a classically activated or inflammatory state, to an alternatively activated or inflammation-resolving state, and to a deactivated state^2^. The dysregulation of macrophage activation and functions appears to be a major contributor to the conversion of acute inflammation into a chronic process^3^ and to the development and progression of atherosclerotic lesions^3^.

Macrophage numbers and functionalities within atherosclerotic lesions and their removal from plaques are controlled by four key processes: recruitment, autophagy, apoptosis, and macrophage polarization. A better understanding of the molecular mechanisms that result in the dysregulation of these processes would therefore be critical for the development of targeted therapies for the prevention and treatment of atherosclerosis and possibly other chronic inflammatory diseases associated with metabolic disorders. Metabolic stress promotes monocyte “priming”, i.e. the dysregulation and hypersensitization of blood monocytes to chemokines, resulting in the over-recruitment of monocyte-derived macrophages to sites of vascular injury and the onset of atherogenesis^4^,^5^. Impairment of macrophage autophagy, a process that protects macrophages from cell damage by degrading damaged and potentially noxious and proinflammatory material, has also been implicated in atherosclerotic lesion progression^6^. In addition, dysregulated macrophage apoptosis and impaired phagocytosis of apoptotic macrophages or “efferocytosis” lead to secondary necrosis and increased inflammation, further promoting plaque progression and destabilization^7^,^8^. Finally, dysregulated macrophage polarization may further promote and accelerate atherogenesis as the persistence of classically activated, pro-inflammatory “M1” macrophages and the delayed or impaired appearance of alternatively activated “M2” macrophages, impairs inflammation resolution, further fueling the atherogenic process^9^,^10^.

Macrophage chemotaxis^4^, autophagy^11^, apoptosis^12^,^13^, and polarization^14^ are mediated by mitogen-activated protein kinase (MAPK) pathways, which in turn are counter-regulated by MAPK phosphatases (MKPs)^15^. MKPs are a family of dual-specificity protein phosphatases that are responsible for the dephosphorylation of both phosphothreonine and phosphotyrosine residues in MAPKs, including JNK, p38 MAPK, and ERK^15^. Dephosphorylation of MAPKs by MKPs inhibits MAPK activity, thereby negatively regulating MAPK signaling.

We recently identified MKP-1 as a critical regulator of monocyte adhesion and migration which, when inactivated and degraded in response to metabolic stress, promotes macrophage recruitment and accumulation in early atherosclerotic lesions^4^. MKP-1 was initially characterized as an ERK1 and ERK2 phosphatase^16^. Subsequent studies indicated that p38 and JNK MAPKs are also MKP-1 substrates and that MKP-1 is capable of inactivating all three classes of MAPK *in vivo*^17^. MKP-1 has been reported to inhibit apoptotic events in leukemia cells^18^, and a recent study suggests that MKP-1 may control macrophage phenotypic transitions during tissue repair^19^. However, little was known about the roles of MKP-1 in macrophage apoptosis, autophagy and polarization. Our study shows that, in addition to promoting macrophage recruitment and accumulation, MKP-1 deficiency in monocytes and macrophages accelerates atherogenesis and lesion progression by impairing macrophage autophagy, enhancing apoptosis, and by dysregulating macrophage polarization whereas overexpression of MKP-1 protects macrophages against metabolic stress-induced dysfunction. Our findings identified MKP-1 as a master regulator of macrophage function and fate.

## Results

### MKP-1 deficiency in macrophages promotes atherosclerosis

**Figure 1:**
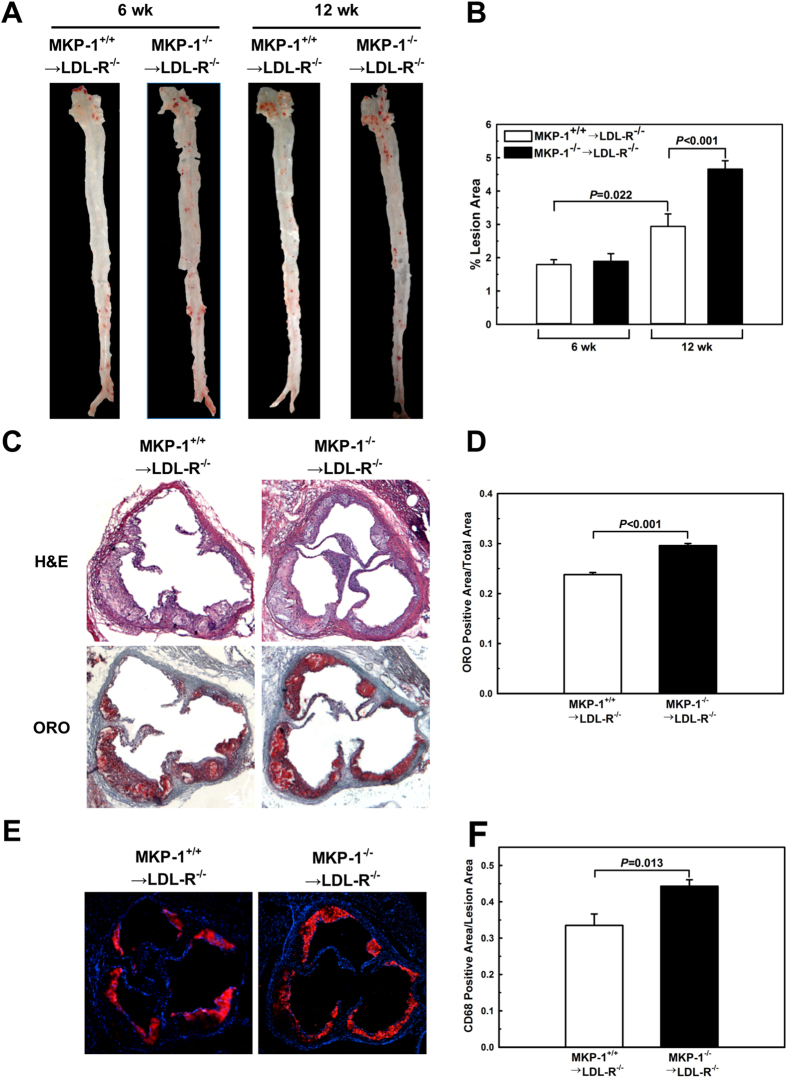
MKP-1 deficiency in macrophages promotes atherosclerosis. Bone marrow transplantation was performed in LDL-R^−/−^ mice using wildtype and MKP-1^−/−^ mice as bone marrow donors. Mice were fed a high-fat diet for 6 and 12 wk. Hearts and aortas of bone marrow recipients were removed, and the size of atherosclerotic lesions was determined by en face analysis (**A+B**) and Oil Red O–stained sections from the aortic roots (**C+D**). Experiments were performed using 5–10 mice per experimental group. Macrophage contents in the atherosclerotic aortic roots was assessed by analysis of CD68-stained sections from the aortic root (**E+F**). Experiments were performed using 6 mice per experimental group. Results shown are mean ± SE.

We showed that hematopoietic MKP-1-deficiency in atherosclerosis-prone mice accelerates atherosclerotic lesion formation^4^, but the underlying mechanisms were not known. To examine the effects of MKP-1 deficiency on macrophage function *in vivo*, we performed bone marrow transplantation experiments in low density lipoprotein receptor-deficient (LDL-R^−/−^) mice using MKP-1^−/−^ and wildtype control mice as bone marrow donors. We induced moderate metabolic stress by feeding the transplant recipients a high-fat diet for six and 12 weeks, and assessed monocyte chemotactic responses *in vivo* and atherosclerotic lesions formation. After only six weeks of HFD feeding, MKP-1 deficient monocytes showed a 4.2-fold higher chemotactic activity in response to monocyte chemoattractant protein-1 (MCP-1) than monocytes in mice that received wildtype bone marrow, indicating that these monocytes were already “primed” and dysfunctional (Suppl. Fig. 1)^4^,^5^. At this early stage, both groups of mice showed small atherosclerotic lesions in both the aortic arch and the descending aorta (Fig. 1A+B), but we did not detect a significant difference in lesion size between the two groups. These findings suggest that monocyte priming and dysfunction induced by MKP-1 deficiency precedes the formation of atherosclerotic lesions.

After 12 weeks on a HFD, the chemotactic response to MCP-1 of MKP-1-deficient monocytes showed no further increase but remained significantly higher (3.3-fold) than that of monocytes in LDL-R^−/−^ recipients of wildtype bone marrow (Suppl. Fig. 1). Atherosclerotic lesion size was increased in both groups, compared to the 6-week time point. However, compared to recipients of wildtype bone marrow, LDL-R^−/−^ mice with MKP-1-deficient bone marrow showed a 1.6-fold increase in atherosclerotic lesion formation as assessed by *en face* analysis (Fig. 1A+B). The acceleration of atherogenesis by hematopoietic MKP-1 deficiency was not due to changes in blood glucose levels and plasma lipids as these mice actually showed slightly lower total plasma cholesterol than mice that received wildtype bone marrow, and plasma triglyceride levels and lipoprotein profiles were not significantly affected by hematopoietic MKP-1 deficiency (Suppl. Fig. 2+3). Monocyte counts were also not different between the two groups (WT: (0.16 ± 0.05) × 10^9^/l versus MKP-1^−/−^: (0.19 ± 0.03) × 10^9^/l; *P* = 0.68).

Histological assessment of atherosclerotic lesions at the aortic sinus after 12 weeks of HFD feeding confirmed the results of the *en* face analysis, revealing a 24% larger foam cell-rich lesion area in mice with hematopoietic MKP-1 deficiency compared to mice that received wildtype bone marrow (Fig. 1C+D). In addition, lesions in mice with hematopoietic MKP-1 deficiency showed a greater CD68-positive area per lesion area (Fig. 1E+F), indicating a higher macrophage content than lesions in control mice. Together these findings suggest that hematopoietic MKP-1-deficiency in atherosclerosis-prone mice accelerates atherogenesis at least in part by sensitizing blood monocytes to chemokines, increasing their chemotactic activity and by enhancing macrophage infiltration and accumulation in atherosclerotic lesions.

### MKP-1-deficient macrophages are autophagy-defective

**Figure 2:**
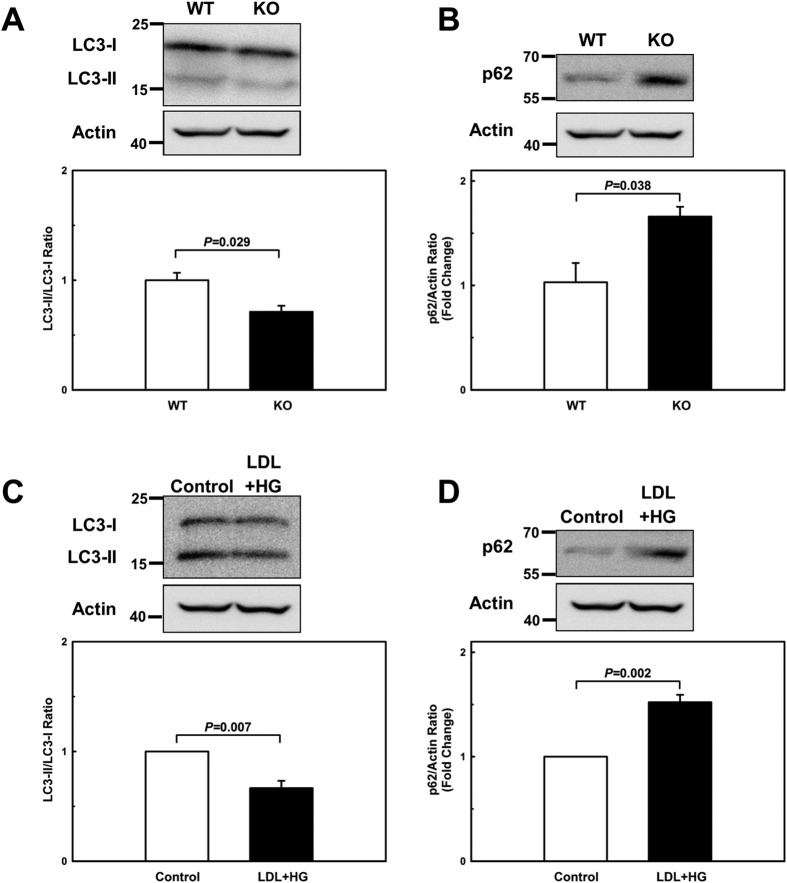
Both MKP-1-deficient and metabolically primed macrophages exhibit defective autophagy. (**A+B**) Autophagic activity was assessed by Western blot analysis as the ratio of LC3-II to LC3-I and p62/SQSTM1 levels in peritoneal macrophages isolated wildtype (WT) and from MKP-1^−/−^ (KO) mice and (**C+D**) in unprimed (Control) and metabolically primed (LDL + HG) peritoneal macrophages. Results shown are mean ± SE of 3 independent experiments.

Autophagy is one of the cell’s survival responses to the stress, and protects macrophages in the plaque by degrading damaged, potentially noxious and proinflammatory material formed within the cells^20^,^21^. Autophagy is regulated by MAPK-dependent pathways^11^,^22^,^23^,^24^. MAPKs are counter-regulated by MKPs, including MKP-1^15^. We therefore examined whether MKP-1 deficiency impairs macrophage autophagy. To this end, we isolated peritoneal macrophages from wildtype and MKP-1^−/−^ mice, and assessed the ratio of light chain 3 (LC3)-II to LC3-I levels as measures of autophagic flux^25^ and p62/ sequestosome 1 (SQSTM1) as an indicator of flux impairment^26^, respectively. As a positive control of the LC3-II to LC3-I ratio shift, peritoneal macrophages were treated with mechanistic target of rapamycin (mTOR) inhibitor (Suppl. Fig. 4). The ratio of LC3-II to LC3-I was decreased by 29% and p62/SQSTM1 levels were increased by 86% in MKP-1-deficient macrophages (Fig. 2A+B), confirming that MKP-1 activity is critical for the maintenance of macrophage autophagy.

Since metabolic stress promotes the *S*-glutathionylation, inactivation and subsequent degradation of MKP-1 in blood monocytes^4^, we next examined whether metabolic stress is sufficient to impair macrophage autophagy. To this end, we metabolically primed peritoneal macrophages from C57BL/6 mice by preincubating the cells for 24 h with human LDL (100 μg/ml) plus high glucose (25 mmol/L)^4^,^5^. We then examined autophagic flux by measuring the ratio of LC3-II to LC3-I and p62/SQSTM1 levels. Metabolic stress reduced the ratio of LC3-II to LC3-I in mouse peritoneal macrophages by 51% (Fig. 2C) and increased p62/SQSTM1 levels 1.5-fold (Fig. 2D), indicating that metabolic stress impairs autophagy in macrophages. As expected, MKP-1 levels were reduced by 39% in these macrophages (Suppl. Fig. 5). Together, these results suggest that monocyte priming induced by metabolic stress, and the associated partial loss of MKP-1 activity, may predispose monocyte-derived macrophages recruited to atherosclerotic lesions to impaired autophagy.

**Figure 3:**
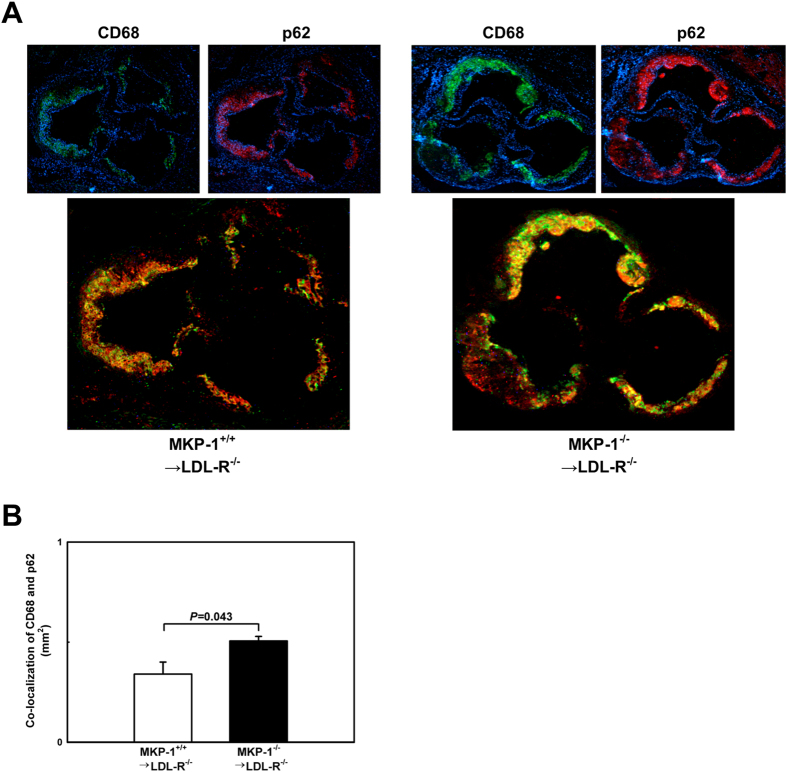
Loss of MKP-1 activity impairs macrophage autophagy in atherosclerotic lesions. (**A+B**) Autophagy in the atherosclerotic aortic roots of mice that received either wildtype or MKP-1^−/−^ bone marrow and were fed a high-fat diet for 12 weeks was assessed by immunofluorescence. The autophagy marker p62/SQSTM1 (red) was concurrently imaged with CD68 (green). Experiments were performed using 5 mice per experimental group. Results shown are mean ± SE.

This mechanistic link between metabolic priming of blood monocytes, loss of MKP-1 activity and impaired macrophage autophagy is further supported by immunofluorescence data we obtained in atherosclerotic lesions from chimeric LDL-R^−/−^ mice that received either wildtype or MKP-1-deficient bone marrow. We found a 49% increase in p62 levels in macrophages in atherosclerotic lesions of LDL-R^−/−^ mice with hematopoietic MKP-1 deficiency compared to mice with MKP-1-expressing macrophages (Fig. 3A+B). This finding confirms our *in vitro* data and strongly suggests that loss of MKP-1 activity impairs macrophage autophagy in atherosclerotic lesions.

### Loss of MKP-1 activity sensitizes oxysterol-induced cell death in macrophages

**Figure 4:**
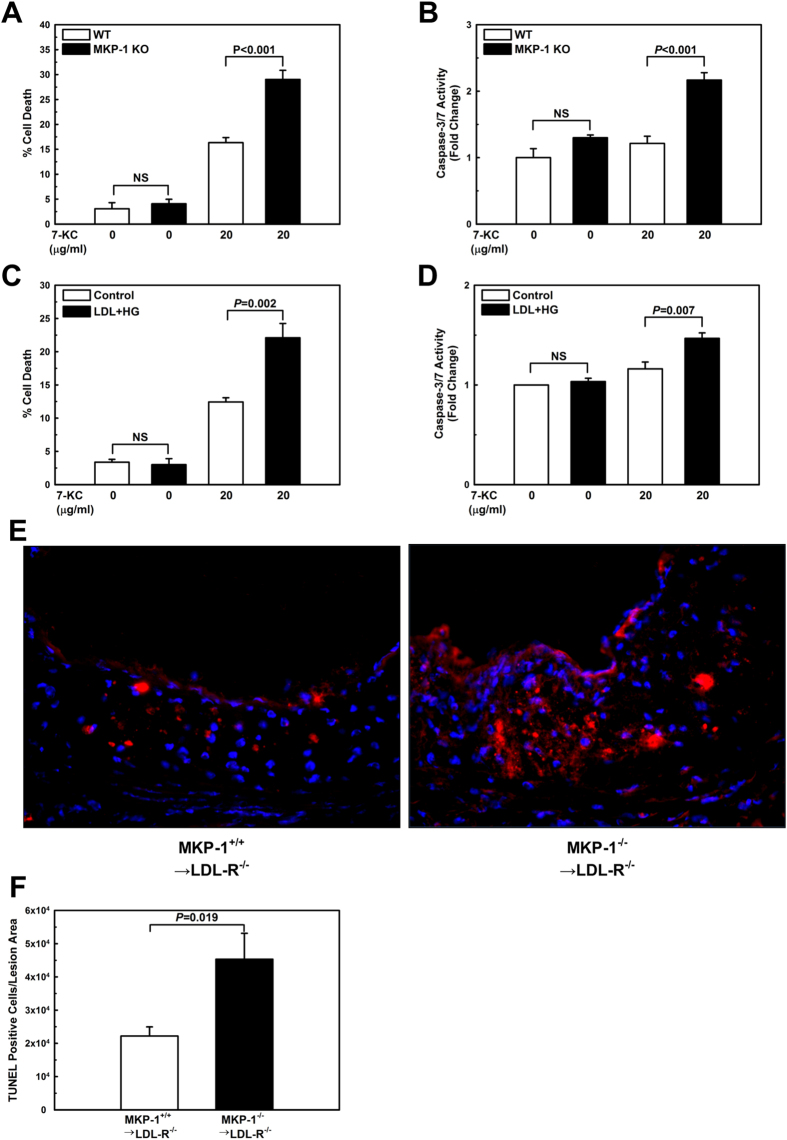
Both MKP-1-deficient and metabolically primed macrophages are sensitized to oxysterol-induced apoptosis. Apoptosis was assessed in peritoneal macrophages treated with vehicle or 7-KC for 24 h. Cell death was measured by trypan blue dye exclusion and caspase 3/7 activation in peritoneal macrophages isolated from wildtype (WT) and MKP-1^−/−^ (KO) mice (**A+B**), and in unprimed (Control) and metabolically primed (LDL + HG) peritoneal macrophages from C57/BL6 mice (**C+D**). Results are shown as mean ± SE (n = 3–4). Cell death in the atherosclerotic aortic roots of mice that received either wildtype or MKP-1^−/−^ bone marrow and were fed a high-fat diet for 12 wk was assessed by TUNEL-positive cells relative to the atherosclerotic lesion area (**E+F**). Experiments were performed using 5–8 mice per experimental group (MKP-1^+/+^ → LDL-R^−/−^: n = 5; MKP-1^−/−^ → LDL-R^−/−^: n = 8). Results are shown as mean ± SE.

Both p38 and JNK MAP kinases play key roles in macrophage apoptosis^8^,^27^,^28^. It is well established that the sustained induction of apoptosis in macrophages within advanced plaques results in a significant increase in lesion size^29^. We therefore examined whether MKP-1 deficiency sensitizes macrophages to apoptosis induced by oxysterols. Since 7-ketocholesterol (7-KC), a major oxidation product of cholesterol found in human atherosclerotic plaque^30^, is cytotoxic to macrophages^31^, we treated peritoneal macrophages isolated from wildtype and MKP-1^−/−^ mice for 24 h with either vehicle or 7-KC to induce apoptosis. Cell death was determined by trypan blue dye exclusion (Suppl. Fig. 6) and by measuring caspase 3/7 activation (Suppl. Fig. 7). Cell death and caspase 3/7 activities in response to 7-KC were markedly increased in MKP-1-deficient macrophages compared to macrophages from wildtype mice (Fig. 4A+B), indicating that MKP-1 protects macrophages against oxysterol-induced cell death. Since metabolic stress results in the loss of MKP-1 activity^4^, we explored whether metabolic stress is sufficient to predispose peritoneal macrophages to oxysterol-induced apoptosis. As expected, metabolic stress had a very similar sensitizing effect on 7-KC-induced cell death as genetic MKP-1 deficiency (Fig. 4C+D).

To investigate whether MKP-1 deficiency also enhances macrophages apoptosis in atherosclerotic lesions, we analyzed terminal deoxynucleotidyl transferase dUTP nick end labeling (TUNEL)-stained aortic root sections from LDL-R^−/−^ mice that received either wildtype or MKP-1-deficient bone marrow. Lesions from LDL-R^−/−^ mice with hematopoietic MKP-1 deficiency contained more than twice as many apoptotic cells per lesion area than lesions from LDL-R^−/−^ mice with MKP-1 expressing macrophages (Fig. 4E+F). These findings suggest that MKP-1 deficiency in macrophages promotes that accumulation of apoptotic cells in atherosclerotic lesions.

### MKP-1 deficiency enhances M1 polarization of macrophages and dampens the conversion of macrophages into an inflammation-resolving M2 phenotype

**Figure 5:**
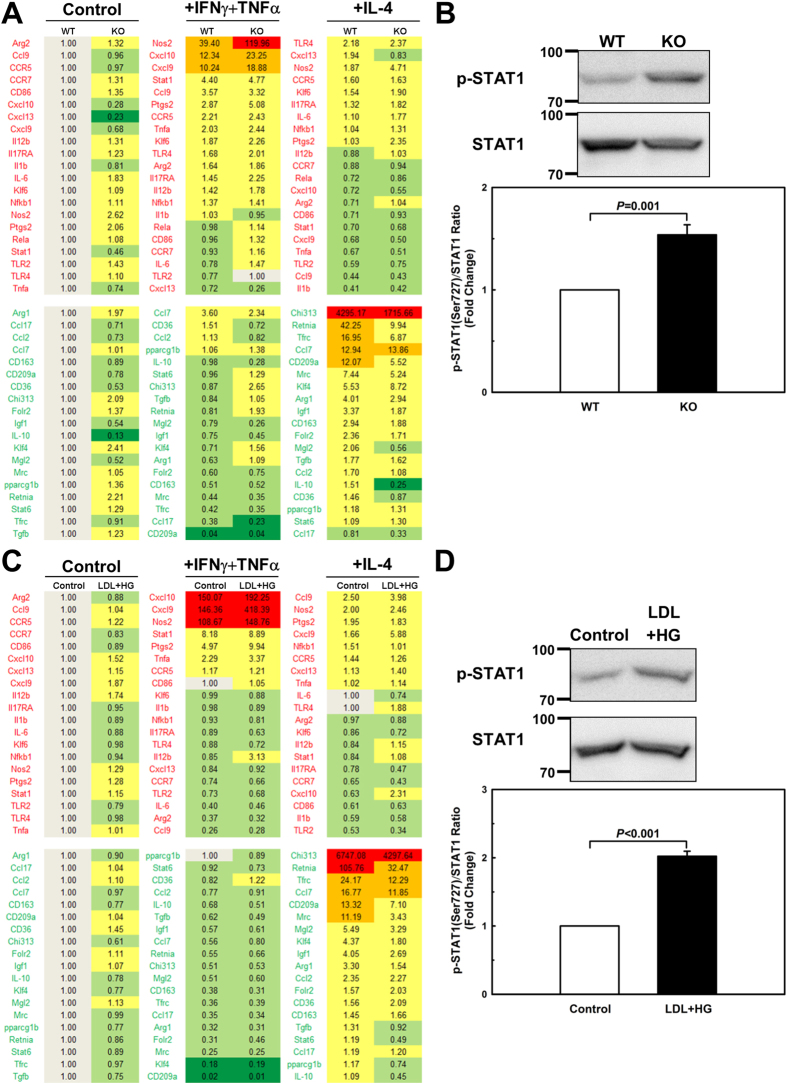
Metabolic priming and MKP-1 deficiency enhance macrophage M1 polarization and suppress the conversion of macrophages into an M2, inflammation-resolving phenotype. Peritoneal macrophages from wildtype (WT) or MKP-1^−/−^ (KO) mice, and unprimed (Control) and metabolically primed (LDL + HG) peritoneal macrophages were polarized as follows: TNF-α plus IFN-γ stimulation to generate M1 macrophages, and IL-4 stimulation as a model of M2 polarization. Untreated peritoneal cells were considered unpolarized. Macrophage polarization was assessed by quantifying and comparing levels of the indicated mRNAs between wildtype and MKP-1^−/−^, and unpirmed and primed macrophages by RT-qPCR. Expression levels were determined using TaqMan^®^ probes in conjunction with the BioMark^TM^ HD Fluidigm System and Fluidigm Real-Time PCR Analysis software. mRNA levels were normalized to a housekeeping gene (*Hprt*) as well as unprimed macrophages (Control), and the 2^−ΔΔCt^ for each mRNA is reported (**A+C**). Results are shown as mean of 4 independent samples. STAT1 phosphorylation were assessed with peritoneal macrophages from wildtype (WT) or MKP-1^−/−^ (KO) mice, and unprimed (Control) and metabolically primed (LDL + HG) peritoneal macrophages (**B+D**). Results are shown as mean ± SE (n = 3–4).

In atherosclerotic lesions, as in other inflamed tissues, the microenvironment plays a key role in determining the activation state of macrophages. Cytokines are an important constituent of the inflammatory milieu and drive macrophage activation either toward a classical (M1, proinflammatory) or alternative (M2, anti-inflammatory) polarization state^3^. The M1 macrophage phenotype is controlled by NF-κB, signal transducer and activator of transcription 1 (STAT1) and interferon-regulatory factor 5 (IRF5)^32^,^33^, but STAT1 activity is essential for macrophages to polarize into an M1 phenotype^33^,^34^. Phosphorylation of STAT1 at Ser727 is required for its activation^35^,^36^. P38 MAPK is required for STAT1 phosphorylation and transcriptional activation induced by interferons^35^,^37^. Interestingly, STAT1 has been suggested as a physiological non-MAPK substrate for MKP-1^38^,^39^. We used real-time PCR in custom-designed microfluidic dynamic arrays^40^,^41^ to explore how MKP-1 deficiency affects macrophage polarization into M1 or M2 activation states. We found that MKP-1 deficiency enhanced interferon gamma+ tumor necrosis factor alpha (INFγ + TNFα)-induced polarization of macrophages toward an M1 phenotype and suppressed the interleukin-4 (IL-4)-induced conversion of macrophages into an M2 phenotype (Fig. 5A). STAT1 phosphorylation was increased by 53% in MKP-1-deficient macrophages as compared to wildtype cells (Fig. 5B), confirming that STAT1, at least in macrophages, is indeed a physiological non-MAPK substrate for MKP-1.

To determine whether metabolic stress again had a similar effect on macrophage polarization as MKP-1 deficiency, we repeated these experiments with peritoneal macrophages isolated from C57BL/6 mice that were metabolically primed, i.e. pre-incubated for 24 h with human LDL (100 μg/ml) plus high glucose (25 mmol/L) prior to being activated with either INFγ + TNFα (M1) or IL-4 (M2). Like MKP-1-deficient macrophages, metabolically primed macrophages displayed enhanced expression of the M1 markers (red font) in response to INFγ + TNFα, but suppressed expression of M2 markers (green font) after IL-4 stimulation (Fig. 5C). Like MKP-1-deficient macrophages, metabolically primed macrophages showed a significantly higher level of STAT1 phosphorylation (202%; Fig. 5D). Together, these findings confirm that metabolically primed macrophages, like MKP-1-deficient macrophages, are pre-programmed for exaggerated M1 polarization but suppressed M2 activation. In both cases, this skewed polarization profile appears to be due to the hyperactivation of STAT1 as a results of reduced MKP-1 activity.

### Overexpression of MKP-1 protects macrophages against impaired autophagy, accelerated cell death, and skewed polarization induced by metabolic stress

**Figure 6:**
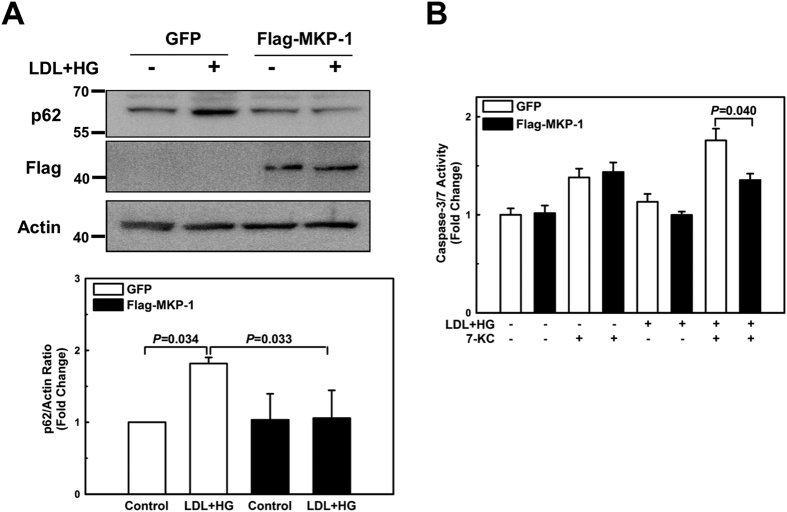
Overexpression of MKP-1 protects macrophages against impaired autophagy, and accelerated cell death. Bone marrow-derived macrophages were infected with lentiviral vectors carrying either Flag-tagged MKP-1 or GFP. Cells were then treated for 24 hours with vehicle or primed with LDL + HG, and p62 protein levels (**A**), Caspase-3/7 activities (**B** Results are shown as mean ± SE (n = 3–4).

**Figure 7:**
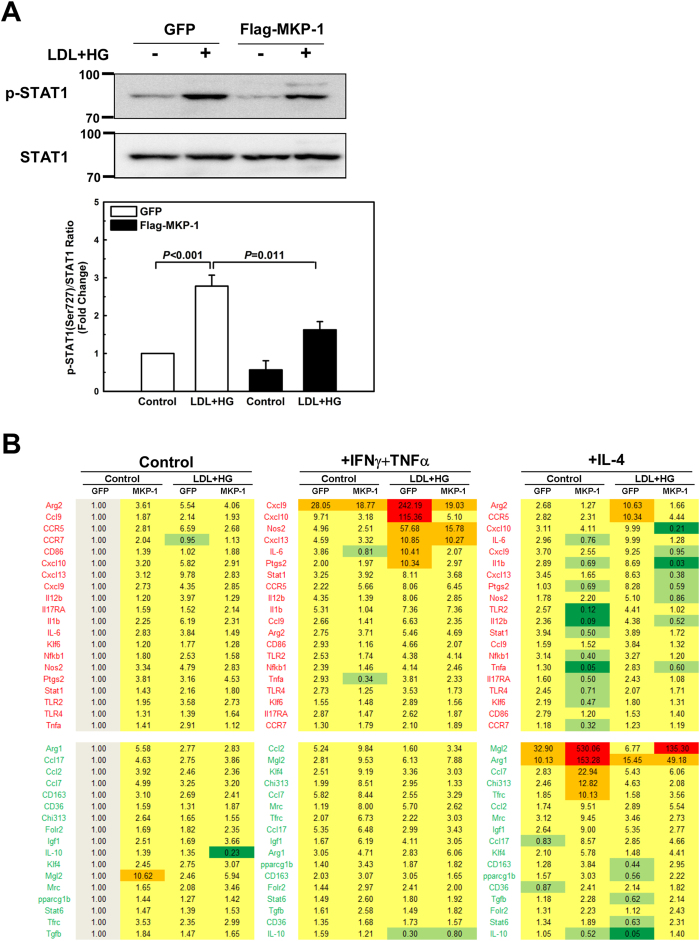
Overexpression of MKP-1 protects macrophages against skewed polarization induced by metabolic stress. Bone marrow-derived macrophages were infected with lentiviral vectors carrying either Flag-tagged MKP-1 or GFP. Cells were then treated for 24 hours with vehicle or primed with LDL + HG, and STAT1 phosphorylation (**A**), and macrophage polarization were assessed (**B**). Results are shown as mean ± SE (n = 3–4).

Metabolic stress promotes MKP-1 inactivation and degradation, and metabolically primed macrophages show all the phenotypic and functional hallmarks of macrophages with genetic MKP-1 deficiency. We therefore determined whether overexpression of MKP-1 protects metabolically stressed macrophages against defective autophagy, sensitization to oxysterol-induced cell death, and skewed M1/M2 polarization. To this end, we used a lentivirus-based transduction system to overexpress Flag-tagged MKP-1 (or green fluorescent protein, GFP) in bone marrow-derived macrophages. In contrast to GFP-expressing control macrophages, macrophages expressing Flag-MKP-1 were resistant to metabolic stress and showed no defects in autophagy (Fig. 6A) or any increased sensitivity to oxysterol-induced activation of caspase 3/7 (Fig. 6B). Furthermore, STAT1 phosphorylation induced by metabolic priming was suppressed in macrophages overexpressing MKP-1 (Fig. 7A) and the skewing of the polarization profile was prevented in MKP-1 overexpressing cells (Fig. 7B). These data provide further evidence that MKP-1 is essential for macrophages to maintain autophagy, resist cytotoxic stress induced by oxysterols and to properly respond to both pro-inflammatory as well as inflammation-resolving signals.

## Discussion

Macrophages are critical for tissue repair as they promote both inflammation, during the early phases, as well as inflammation resolution critical for wound healing^42^. Coordinating these complex functions requires both a high degree of plasticity as well as a tight regulation of cell signaling and cellular functions.

We identified MKP-1 as a regulator of monocyte signaling, activation and function, sensitive to metabolic stress-induced inactivation and *S*-glutathionylation dependent degradation^4^. We therefore hypothesized that dysregulation and loss of MKP-1 activity in blood monocytes induced by metabolic disorders may have profound effects on macrophages derived from these monocytes, which are recruited to sites of tissue injury and may affect macrophage functions associated with both inflammatory as well as inflammation resolving processes. Thus, loss of MKP-1 in macrophages may play a central role in chronic inflammatory diseases such as atherosclerosis. We found that genetic MKP-1 deficiency impairs macrophage autophagy, sensitizes macrophages to oxysterol-induced cell death, and enhances M1 polarization while suppressing M2 polarization. We also provide direct evidence that metabolic stress-induced MKP-1 deficiency in blood monocytes directly promotes atherogenesis by enhancing monocyte recruitment into the vessel wall, dysregulating macrophage plasticity and by predisposing monocyte-derived macrophages for impaired autophagy and increased cell death within atherosclerotic lesions.

MKP-1 preferentially dephosphorylates activated p38 MAPK and JNK relative to ERK^43^. Studies with MKP-1 knockout mice provided compelling evidence that MKP-1 is an important regulator of innate and adaptive immune responses, inflammation and infection control by regulating p38 MAPK and JNK activation in response to stimuli and stress^44^,^45^. Additionally, MKP-1-deficient fibroblasts exhibit enhanced sensitivity to apoptosis mediated by p38 MAPK hyperactivation, suggesting a role for MKP-1 in cell survival signaling^46^. Our finding that MKP-1 deficient macrophages in culture are significantly more sensitive than wildtype macrophages to inducers of apoptosis, such as oxysterols, supports that hypothesis. The fact that MKP-1-deficiency sensitized macrophages to 7-KC-induced apoptosis and that atherosclerotic lesions of recipients of MKP-1-deficent bone marrow showed a dramatic increase in apoptotic macrophages strongly suggests that MKP-1 plays a critical role in protecting macrophages in atherosclerotic plaques against oxysterol-induced cell injury and death.

To date, MKP-1 had not been linked to autophagy, however, p38 MAPK activation was shown to inhibit autophagy in macrophages^11^ and in human CD8^+^ T cells^47^, which suggested that MKP-1 may also play an important role in macrophage autophagy. Indeed, we found that MKP-1 deficiency significantly impairs autophagy and autophagic flux. Considering the pro-survival role of autophagy^48^,^49^, impaired autophagy may have at least partly contributed to the increased vulnerability of MKP-1-deficient macrophages to oxysterol-induced apoptosis we observed both *in vitro* and *in vivo* (Fig. 4).

Polarization of macrophages into either classically activated M1 or alternatively activated M2 macrophages is regulated by STAT and MAPK pathways. Here we provide the first evidence that MKP-1 plays a critical role in regulating macrophage polarization, and that loss of MKP-1 activity results in dysregulated macrophage activation and polarization. STAT1 is a key activator of M1 macrophage polarization, and Ser727 phosphorylation is essential for M1 macrophage polarization^35^,^36^,^50^,^51^. Importantly, p38 MAPK is required for STAT1 activation^35^,^37^. Moreover, STAT1 has been suggested to be a physiological non-MAPK substrate for MKP-1^38^,^39^ and we observed that MKP-1 inhibition, either by MKP-1 deficiency or metabolic stress, increased STAT1 phosphorylation at Ser727. This finding suggests that reduced MKP-1 activity results in the hyperactivation of STAT1 (Fig. 5B,D), and this mechanism would account for the hyper-inflammatory profile of M1 (IFNγ + TNFα) polarized macrophages that were exposed to metabolic stress (Fig. 5C). We therefore provide evidence that STAT1 in macrophages is in fact a physiological non-MAPK substrate for MKP-1.

Previously, we showed that metabolic stress decreases MKP-1 levels and activity in monocytes and macrophages, and both the inactivation and degradation of MKP-1 are mediated by the *S*-glutathionylation of the catalytic cysteine residue 258^4^. Here, we show that metabolic stress mimics the effects of genetic MKP-1 deficiency, including defective autophagy, sensitization to oxysterol-induced cell death, and skewed M1/M2 polarization. We demonstrated that MKP-1 deficiency occurs in blood monocytes of diabetic mice^4^. Oxidative stress induced by chronic metabolic stress is likely to cause macrophage MKP-1 deficiency, and macrophages deficient in MKP-1 are likely to be found in microenvironments with high metabolic stress, such as those found in atherosclerotic lesions (Fig. 1E) and lipid-enriched diabetic environments. If these microenvironments persist and become chronic, they are likely to promote local macrophage dysfunction, which, in turn, is likely to increase inflammation and impair inflammation resolution at those sites.

Overexpression of MKP-1 not only prevents monocyte priming and hypersensitization to chemokines^4^, but also protected metabolically stressed macrophages against defective autophagy, sensitization to oxysterol-induced cell death, and skewed M1/M2 polarization. These results provide further evidence that MKP-1 is a key regulator of monocyte and macrophage function, activation, and plasticity, and that MKP-1 deficiency induced by metabolic stress, promotes macrophage dysfunction, a process that may play a fundamental role in the transition of acute inflammation to states of chronic inflammation typically associated with metabolic disorders. These findings also suggest that therapeutic or pharmacological strategies aimed at preserving monocyte and macrophage MKP-1 activity are likely to protect against chronic inflammatory disease associated with metabolic disorders.

Another intriguing possibility is that MKP-1 deficiency occurs early on during macrophage development, i.e. in circulating monocytes exposed to hyperglycemia and dyslipidemia, or even in as early as during myelopoiesis, and leads to the epigenetic reprogramming of blood monocytes. MKP-1 is the only known mammalian histone H3 serine 10 phosphatase^52^. It is well-known that during mitosis serine 10 on histone H3 is highly phosphorylated on condensed chromosomes^53^,^54^, and phosphorylated serine 10 has therefore been used as mitotic marker. However, recent studies have demonstrated that a diverse array of stimuli are able to rapidly induce phosphorylation of serine 10 on histone H3, a process that is associated with transcriptional activation of immediate early genes, such as c-fos and c-jun^55^,^56^. Phosphorylation of serine 10 on histone H3 is also known to mediate expression of genes related to inflammation such as cyclooxygenase (COX)-2^57^, interleukin (IL)-6, and IL-8^58^,^59^. Therefore, it is conceivable that MKP-1 deficiency induced by metabolic stress leads to epigenetic changes in blood monocytes, at least in part mediated through serine 10 phosphorylation on histone H3 (Suppl. Fig. 8). Epigenetic reprogramming of blood monocytes in response to chronic metabolic stress could account for the dysregulation of monocyte-derived macrophages associated with chronic inflammation and impaired wound healing, hallmarks of metabolic disorders such as hyperlipidemia and diabetes.

In summary, we discovered a new mechanistic link between metabolic disorders, macrophage dysfunction, and atherogenesis. With the identification of macrophage MKP-1 as an early sensor of metabolic stress and a master regulator of macrophage autophagy, apoptosis, and polarization, and loss of MKP-1 activity as a key mechanism for macrophage dysfunction induced by metabolic stress, both *in vitro* and *in vivo*, we may also have identified a novel biomarker and potential therapeutic target for the prevention and treatment of atherosclerosis.

## Methods

Detailed methods are described in the Supplemental Materials.

### Animals and diets

Female LDL-R^−/−^ recipient mice and female C57BL/6 donor mice were obtained from Jackson Labs (Bar Harbor, ME). Female MKP-1^−/−^ donor mice were kindly provided by the laboratory of Dr. Robert Kramer. All mice had been backcrossed to C57BL/6 background for more than 10 generations. To induce hypercholesterolemia, bone marrow recipient mice were switched to a high fat diet (HFD; 21% milk fat and 0.2% cholesterol, diet no. F5540, Bio-Serv, Frenchtown, NJ). Mice were maintained on HFD for 6 or 12 weeks. All studies were performed in accordance with the guidelines and regulations of and with the approval of the UTHSCSA Institutional Animal Care and Use Committee.

### Bone Marrow Cell Collection

On the day of bone marrow transplantation, bone marrow cell suspensions were collected from C57BL/6 and MKP-1^−/−^ mice.

### Irradiation and Bone Marrow Transplantation

LDL-R^−/−^ recipient mice were maintained on antibiotics for the duration of the study. Before transplantation, recipient LDL-R^−/−^ mice received 2 equal doses of 4.7 Gy, with 3 h between each dose (9.4 Gy total, Cobalt-60 Irradiator). Animals were given a 4 h recovery period prior to bone marrow transplantation. LDL-R^−/−^ mice were divided into two groups based on the strain of their donors (n = 30/group). Bone marrow cells (10–15 × 10^6^ cells in 150–300 μl) were then injected via the retro-orbital sinus. Animals were given a 4 weeks of recovery prior to initiation of HFD feeding.

### Plasma Cholesterol, Triglycerides, and Lipoprotein Profile

Mice were fasted overnight prior to euthanasia and blood was collected by cardiac puncture. Plasma cholesterol and triglycerides were quantified using enzymatic assay kits per manufacturer’s protocol (Wako Chemicals USA, Inc., Richmond, VA). Plasma was pooled from each treatment group and centrifuged to clarify the sample and 100 μl of pooled plasma was used for size exclusion chromatography.

### *In Vivo* Macrophage Recruitment Assay

Each mouse received two Matrigel plugs three days prior to euthanasia as described previously^5^.

### Histological and Immunohistochemical Analyses of Heart, Aorta, and Aortic Root

After peritoneal lavage, the entire aorta with the intact heart was dissected free of fat and removed. The aortas (proximal ascending aorta to the bifurcation) were fixed in 4% PFA for 48 h prior to staining for *en face* analysis. To determine the extent of the atherosclerosis, aortas were stained with Oil Red O (ORO; Sigma-Aldrich, St. Louis, MO). To further characterize lesions, neutral and intracellular lipid content were quantified by staining sections of heart tissue with ORO. Sections of aortic root were blocked with 10% normal Goat serum and incubated overnight at 4 °C with rat anti-mouse CD68:biotin (1:200; Bio-Rad Laboratories, Inc., Raleigh, NC) primary antibody. Tissues were also stained with antibodies to detect Signaling adaptor p62 (1:200; SQSTM1, Pierce, Rockford, IL), LC3B (1:200; Pierce, Rockford, IL), and the phosphorylation status of signal transducer and activator of transcription 1 (STAT1) at Ser 727 [Phospho-Stat1 (Ser727); 1:100; Cells Signaling Technology, Boston, MA)]. Selected sections of the aortic root were used for the detection of apoptotic and necrotic cells in atherosclerotic lesions. Tissues were analyzed by terminal dUTP terminal nick end-labeling (TUNEL assay; Roche Diagnostics, Indianapolis, IN) according to the manufacturer’s instruction.

### Cell Culture

Resident peritoneal macrophages were collected from C57BL/6 and MKP-1^−/−^ mice by lavage. Adherent macrophages were cultured in RPMI complete supplemented with 10% fetal bovine serum. Bone marrow-derived macrophages (BMDMs) were generated from C57BL/6 mice. Fresh bone marrow cells were cultured for 7 days in RPMI complete medium supplemented with 10% FBS and 50 ng/ml M-CSF. Metabolic stress was induced by incubating macrophages for 24 h with 100 μg/ml LDL and 20 mmol/L D-glucose (LDL + HG). M1 polarization was induced with IFN-γ plus TNF-α (PeproTech; 50 and 10 ng/ml, respectively)^60^. Alternative (M2) polarization was induced by stimulating cells for 24 h with IL-4 (PeproTech; 10 ng/ml)^60^. Macrophages that were grown in the absence of the above cytokines were considered nonpolarized or M0.

### Lentiviral Transduction of Macrophages

All lentiviral supernatants were prepared by cotransfection of HEK-293T cells with one of the vector transfer constructs, murine ecotropic envelope vector (pCAG-Eco, Addgene Plasmid # 35617), and the packaging vectors pMDLg/pRRE and RSV-Rev. For gene transduction, bone marrow-derived macrophages were used on day 5 of differentiation. Two million cells were plated in 12-well plates, and viruses (MOI = 20) were added to each well in the presence of 6 μg/ml of DEAE-dextran sulfate (Sigma-Aldrich, St. Louis, MO).

### Gene Expression

Total RNA was isolated from cells using PureLink RNA Mini Kit (Ambion, Grand Island, NY). Reverse transcription was performed using a QuantiTect Reverse Transcription Kit (Qiagen, Valencia, CA). Each cDNA sample was then separated into 48 separate reactions for qPCR analysis using the BioMark 48×48 dynamic array nanofluidic chip (Fluidigm Inc., USA) according to manufacturer’s instruction. The 40 individual Taqman primer-probe mixtures (Applied Biosystems) specific for individual transcripts of interest are listed in Table S1. Amplification data were analyzed using SDS2.4 software (Applied Biosystems, Grand Island, NY), and gene expression levels were normalized to *Hprt* as the housekeeping gene.

### Statistics

Data were analyzed using ANOVA (Sigma Stat 12.0). Data were tested for use of parametric or nonparametric post hoc analysis, and multiple comparisons were performed by using the Least Significant Difference method. All data are presented as mean ± SE of at least 3 independent experiments. Results were considered statistically significant at the *P* < 0.05 level.

## Additional Information

**How to cite this article**: Kim, H. S. *et al*. Monocytic MKP-1 is a Sensor of the Metabolic Environment and Regulates Function and Phenotypic Fate of Monocyte-Derived Macrophages in Atherosclerosis. *Sci. Rep.* **6**, 34223; doi: 10.1038/srep34223 (2016).

## Supplementary Material

Supplementary Information
