# Supplementary material for: Monocytic MKP-1 is a Sensor of the Metabolic Environment and Regulates Function and Phenotypic Fate of Monocyte-Derived Macrophages in Atherosclerosis
Source: Sci Rep. 2016 Sep 27;6:34223. doi: 10.1038/srep34223 (PMC5037453; doi:10.1038/srep34223)
Supplement: Supplementary Information [file srep34223-s1.doc]

**Monocytic MKP-1 is a Sensor of the Metabolic Environment and Regulates Function and Phenotypic Fate of Monocyte-Derived Macrophages in Atherosclerosis**

Hong Seok Kim^1,2^, Sina Tavakoli^3^, Leigh Ann Piefer^4^, Huynh Nga Nguyen^5^, and Reto Asmis^3,4,5^,*

^1^ Department of Molecular Medicine, College of Medicine, Inha University, Incheon 22212, Republic of Korea

^2^ Hypoxia-related Disease Research Center, College of Medicine, Inha University, Incheon 22212, Republic of Korea

^3^ Department of Radiology, University of Texas Health Science Center at San Antonio

^4^ Department of Clinical Laboratory Sciences, University of Texas Health Science Center at San Antonio

^5^ Department of Biochemistry, University of Texas Health Science Center at San Antonio

* Correspondence should be addressed to: Reto Asmis, PhD, Clinical Laboratory Sciences, School of Health Professions, University of Texas Health Science Center at San Antonio, 8403 Floyd Curl Drive, MC 8254, San Antonio, TX 78229-3904;

Tel.: (210) 562-4054, FAX: (210) 562-4121; email: asmis@uthscsa.edu

**SUPPLEMENTAL INFORMATION**


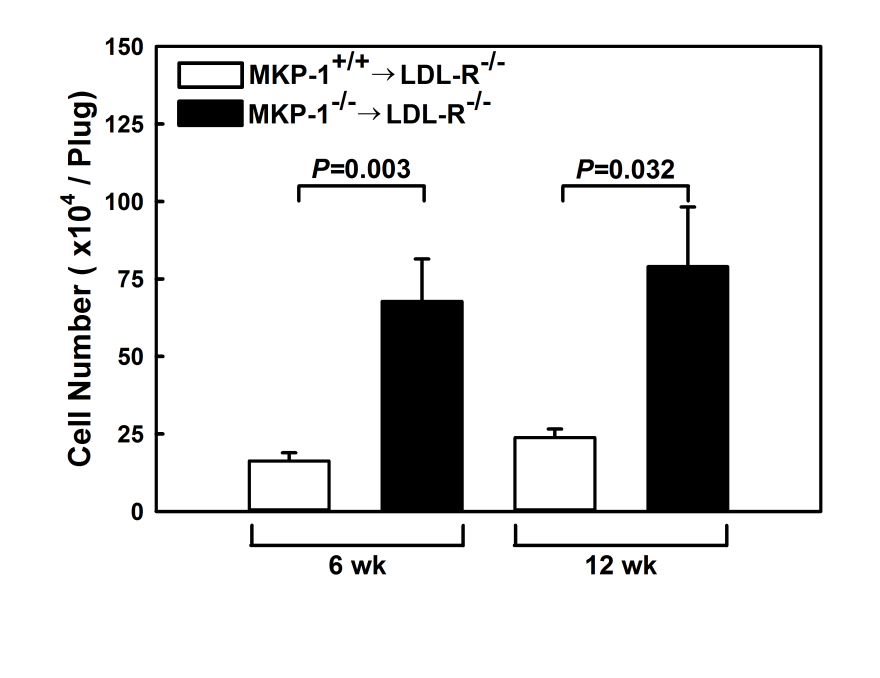


**Figure S1: Hematopoietic MKP-1 deficiency increases monocyte chemotaxis and macrophage recruitment into MCP-1-loaded Matrigel plugs.** Bone marrow transplantation was performed in LDL-R-null mice using wildtype (MKP-1^+/+^) and MKP-1 deficient (MKP-1^-/-^) mice as bone marrow donors. After 6 or 12 weeks on a HFD, Matrigel supplemented with either vehicle or MCP-1 (500 ng/mL) was injected into the left and right flank, respectively, of bone marrow recipients. After three days, Matrigel plugs were surgically removed, dissolved and macrophage content was determined in a fluorescent-based cell counter as described under “Materials and Methods”. Results were calculated for each mouse as the difference in macrophage numbers between MCP-1 and vehicle loaded plugs, and shown as mean ± SE (n=7-10).

**
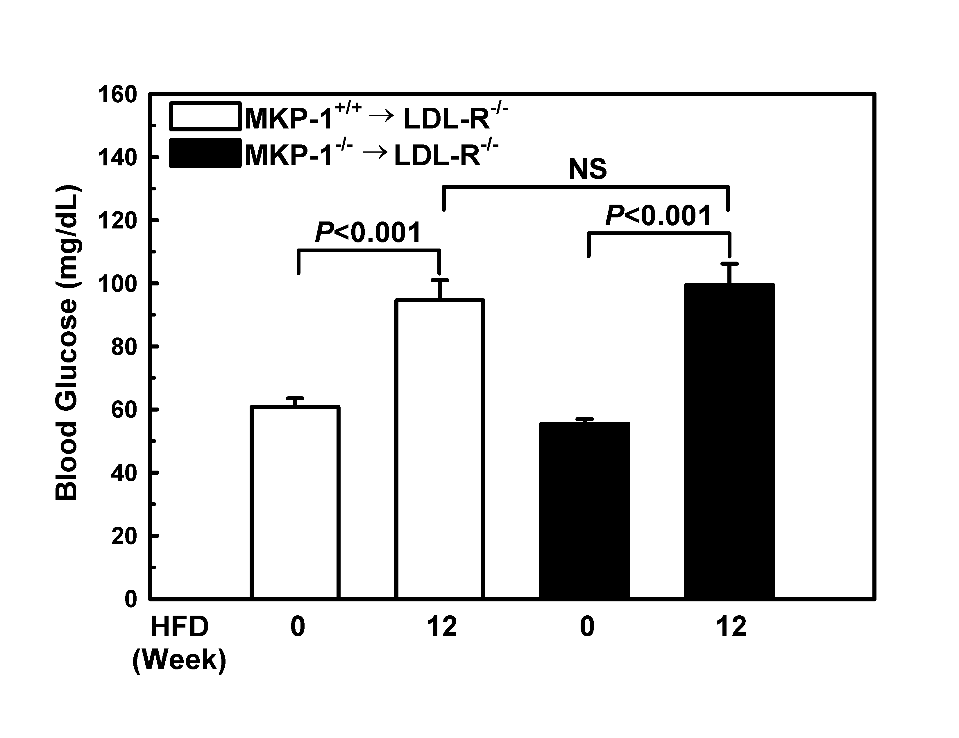
**

**Figure S2: Blood glucose levels of bone marrow recipients.** Fasted blood glucose levels were determined as described in Materials and Methods. Values are mean ± SE for 10-15 mice. NS: not significant

**Figure S3: Plasma lipids of bone marrow recipients.** Fasted total plasma cholesterol (**A**), triglyceride levels (**B**) and FPLC lipoprotein profiles (**C**) were determined as described in Materials and Methods when mice were killed. Results are expressed as means ± SE; n = 5 for wildtype (MKP-1^+/+^) mice; n = 4 for MKP-1 deficient (MKP-1^-/-^) mice.


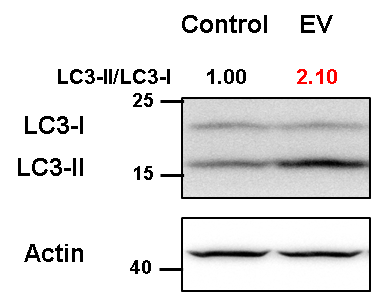


**Figure S4. mTOR inhibitor everolimus increases LC3-II:LC3-I ratio in macrophages.** Peritoneal macrophages were treated with vehicle (control) or everolimus (EV, 100nM) for 24h. LC3 levels were assessed by Western blot analysis. The representative experiment is shown.

**Figure S5. Metabolic priming decreases MKP-1 protein levels in macrophages.** MKP-1 levels were assessed by Western blot analysis in unprimed (C, Control) and metabolically primed (LDL+HG) peritoneal macrophages. Results are shown as mean ± SE (n = 4).


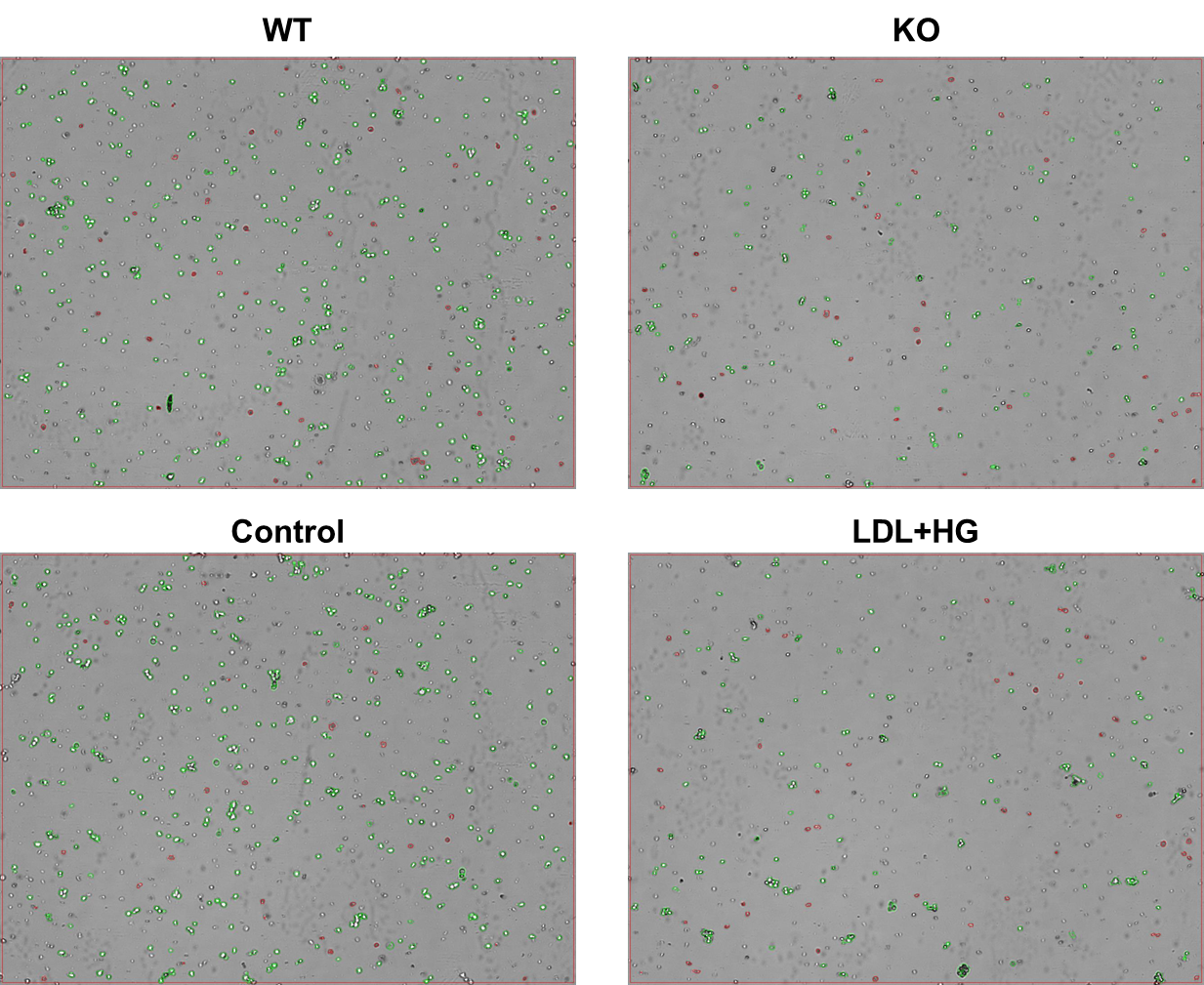


**Figure S6. Both MKP-1-deficient and metabolically primed macrophages are sensitized to oxysterol-induced loss of membrane integrity.** Cell death was measured by trypan blue dye exclusion in peritoneal macrophages treated with vehicle or 7-KC for 24 h from wildtype (WT) and MKP-1^-/-^ (KO) mice, and in unprimed (Control) and metabolically primed (LDL+HG) peritoneal macrophages from C57/BL6 mice. Live cells with a bright center are circled in green and dead cells which are dark and circled in red. Representative experiments are shown.


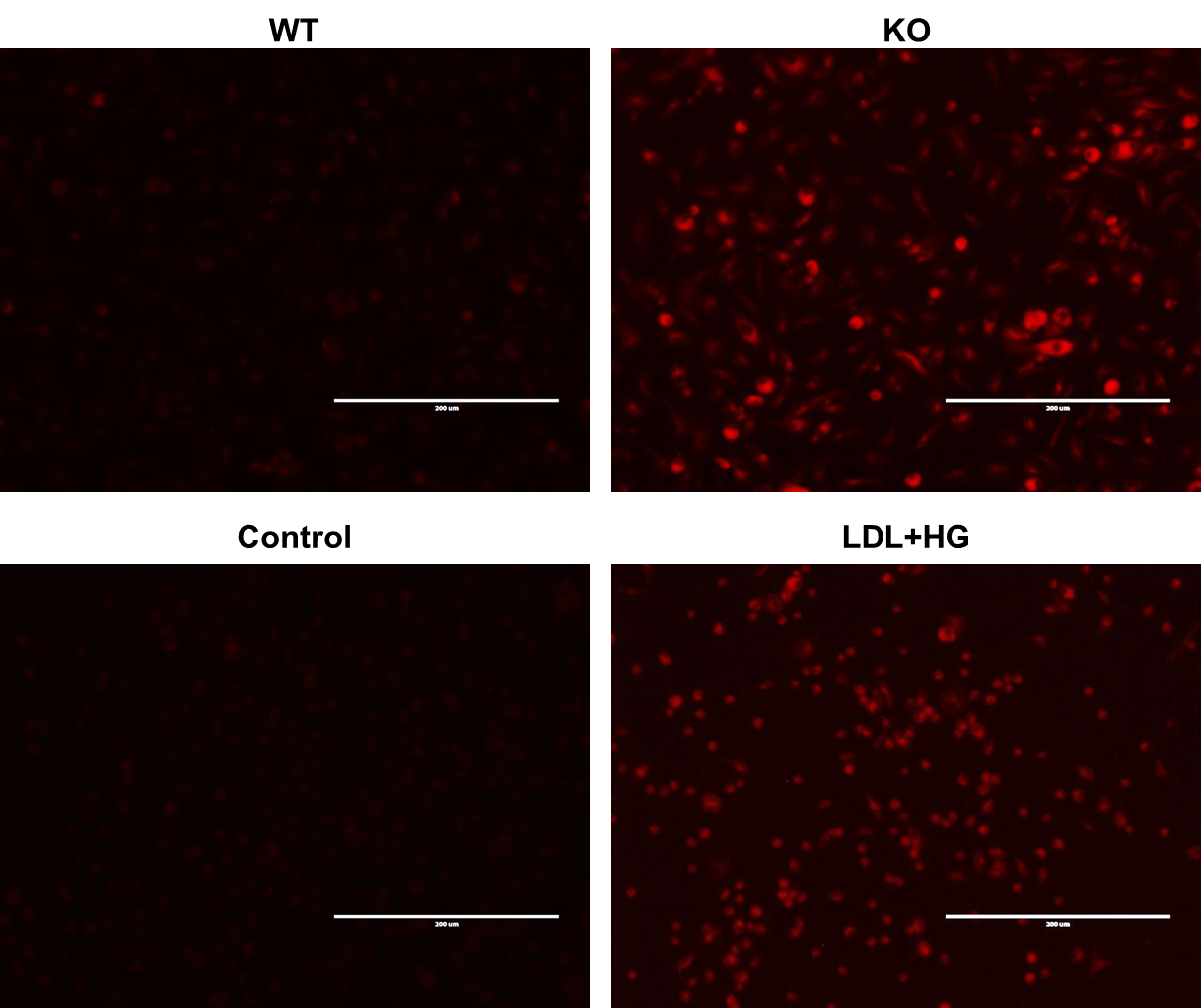

**Figure S7. Both MKP-1-deficient and metabolically primed macrophages are sensitized to oxysterol-induced apoptosis.** Caspase 3/7 activation was measured in peritoneal macrophages treated with vehicle or 7-KC for 24 h from wildtype (WT) and MKP-1^-/-^ (KO) mice, and in unprimed (Control) and metabolically primed (LDL+HG) peritoneal macrophages from C57/BL6 mice. Representative experiments are shown.

**Figure S8: Metabolic priming increases histone H3 phosphorylation at serine 10 residue in monocytes.** THP-1 monocytes were treated for 24 h with vehicle or metabolically primed with LDL+HG. Histone H3 phosphorylation (Ser10) was assessed by Western blot analysis. Two representative experiments are shown.

Table S1. Genes of interest used in this study for mRNA quantification

| **N** | **Gene symbol** | **Gene name** | **Reference sequence** | **Assay ID** |
| --- | --- | --- | --- | --- |
| 1 | Arg2 | arginase type II | NM_009705.3 | Mm00477592_m1 |
| 2 | Ccl9 | chemokine (C-C motif) ligand 9 | NM_011338.2 | Mm00441260_m1 |
| 3 | CCR5 | chemokine (C-C motif) receptor 5 | NM_009917.5 | Mm01963251_s1 |
| 4 | CCR7 | chemokine (C-C motif) receptor 7 | 2 Refseqs | Mm99999130_s1 |
| 5 | CD86 | CD86 antigen | NM_019388.3 | Mm00444543_m1 |
| 6 | Cxcl10 | chemokine (C-X-C motif) ligand 10 | NM_021274.2 | Mm00445235_m1 |
| 7 | Cxcl13 | chemokine (C-X-C motif) ligand 13 | NM_018866.2 | Mm04214185_s1 |
| 8 | Cxcl9 | chemokine (C-X-C motif) ligand 9 | NM_008599.4 | Mm00434946_m1 |
| 9 | Il12b | interleukin 12b | 9 Refseqs | Mm01288989_m1 |
| 10 | Il17RA | interleukin 17 receptor A | NM_008359.2 | Mm00434214_m1 |
| 11 | Il1b | interleukin 1 beta | NM_008361.3 | Mm00434228_m1 |
| 12 | IL-6 | interleukin 6 | NM_031168.1 | Mm00446190_m1 |
| 13 | Nfkb1 | nuclear factor of kappa light polypeptide gene enhancer in B cells 1, p105 | NM_008689.2 | Mm00476361_m1 |
| 14 | Nos2 | nitric oxide synthase 2, inducible | NM_010927.3 | Mm00440502_m1 |
| 15 | Ptgs2 | prostaglandin-endoperoxide synthase 2 | NM_011198.3 | Mm00478374_m1 |
| 16 | Rela | v-rel reticuloendotheliosis viral oncogene homolog A (avian) | NM_009045.4 | Mm00501346_m1 |
| 17 | Stat1 | signal transducer and activator of transcription 1 | 3 Refseqs | Mm00439531_m1 |
| 18 | TLR2 | toll-like receptor 2 | NM_011905.3 | Mm01213946_g1 |
| 19 | TLR4 | toll-like receptor 4 | NM_021297.2 | Mm00445273_m1 |
| 20 | Tnfa | tumor necrosis factor | 2 Refseqs | Mm00443258_m1 |
| 21 | Arg1 | arginase, liver | NM_007482.3 | Mm00475988_m1 |
| 22 | Ccl17 | chemokine (C-C motif) ligand 17 | NM_011332.3 | Mm01244826_g1 |
| 23 | Ccl2 | chemokine (C-C motif) ligand 2 | NM_011333.3 | Mm00441242_m1 |
| 24 | Ccl7 | chemokine (C-C motif) ligand 7 | NM_013654.3 | Mm00443113_m1 |
| 25 | CD163 | CD163 antigen | 2 Refseqs | Mm00474091_m1 |
| 26 | CD209a | CD209a antigen | NM_133238.5 | Mm00460067_m1 |
| 27 | CD36 | CD36 antigen | 5 Refseqs | Mm00432403_m1 |
| 28 | Chi3l3 | chitinase-like 3 | NM_009892.2 | Mm00657889_mH |
| 29 | Folr2 | folate receptor 2 (fetal) | NM_008035.2 | Mm00433357_m1 |
| 30 | Igf1 | insulin-like growth factor 1 | 5 Refseqs | Mm00439560_m1 |
| 31 | IL-10 | interleukin 10 | NM_010548.2 | Mm01288386_m1 |
| 32 | Klf4 | Kruppel-like factor 4 (gut) | NM_010637.3 | Mm00516104_m1 |
| 33 | Mgl2 | macrophage galactose N-acetyl-galactosamine specific lectin 2 | NM_145137.2 | Mm00460844_m1 |
| 34 | Mrc | mannose receptor, C type 1 | NM_008625.2 | Mm01329362_m1 |
| 35 | Ppargc1b | peroxisome proliferative activated receptor, gamma, coactivator 1 beta | NM_133249.2 | Mm00504730_m1 |
| 36 | Retnla | resistin like alpha | NM_020509.3 | Mm00445109_m1 |
| 37 | Stat6 | signal transducer and activator of transcription 6 | NM_009284.2 | Mm01160477_m1 |
| 38 | Tfrc | transferrin receptor | NM_011638.4 | Mm00441941_m1 |
| 39 | Tgfb | transforming growth factor, beta 1 | NM_011577.1 | Mm01178820_m1 |
| 40 | Hprt | hypoxanthine guanine phosphoribosyl transferase | NM_013556.2 | Mm03024075_m1 |

**SUPPLEMENTAL Experimental PROCEDURES**

**Animals and diets**

Female LDL-R^-/-^ recipient mice (B6.129S7-Ldlr^tm1her^/J, stock no. 002207) and female C57BL/6 (stock no. 000664) donor mice were obtained from Jackson Labs (Bar Harbor, ME). Female MKP-1^-/-^ donor mice were kindly provided by the laboratory of Dr. Robert Kramer. All mice had been backcrossed to C57BL/6 background for more than 10 generations, and were maintained in colony cages on a 12-h light/12-h dark cycle and fed a normal mouse laboratory diet. To induce hypercholesterolemia, bone marrow recipient mice were switched to a high fat diet (HFD; 21% milk fat and 0.2% cholesterol, diet no. F5540, Bio-Serv, Frenchtown, NJ) four weeks after bone marrow transplantation (BMT). Mice were maintained on HFD for 6 or 12 weeks. Fasted (overnight) body weight and blood glucose were measured before HFD initiation and after 6 and 12 weeks of HFD feeding. All studies were performed in accordance with the guidelines and regulations of and with the approval of the UTHSCSA Institutional Animal Care and Use Committee.

**Bone Marrow Cell Collection**

On the day of bone marrow transplantation, bone marrow cell suspensions were collected from C57BL/6 and MKP1^-/-^ mice. Donor mice were euthanatized and the entire legs were dissected. Femurs and tibias were removed and cleaned of all adipose and muscle tissue. Bone marrow cells were flushed from the bones using Iscove’s Modified Dulbecco’s Medium (IMDM; Invitrogen, Grand Island, NY) supplemented with 10% heat inactivated fetal bovine serum (FBS; Invitrogen, Grand Island, NY) and 1% penicillin-streptomycin solution (p/s; Corning, Manassas, VA), here on referred to as IMDM-FBS-p/s. Bone marrow cells were pooled from animals of the same strain (n=4/group) and washed twice with IMDM-FBS-p/s before centrifugation. Between washes, cells were passed through a cell strainer to clarify the sample. After washing, the cells were resuspended in IMDM supplemented with 1% p/s (without FBS) and placed on ice until bone marrow injection.

**Irradiation and Bone Marrow Transplantation**

One week prior to irradiation and BMT, LDL-R^-/-^ recipient mice received water supplemented with antibiotics (240 mg sulfamethoxazole-trimethoprim/ 400 ml H_2_O; Hi Tech Pharmacal Co. Inc., Amityville, NY). Mice were maintained on antibiotics for the duration of the study. Before transplantation, recipient LDL-R^-/-^ mice received 2 equal doses of 4.7 Gy, with 3 h between each dose (9.4 Gy total, Cobalt-60 Irradiator). Animals were given a 4 h recovery period prior to bone marrow transplantation.

LDL-R^-/-^ mice were divided into two groups based on the strain of their donors (n=30/group). Prior to injection, mice were anesthetized for 1-2 min under isoflurane anesthesia, ensuring the loss of righting reflexes. Bone marrow cells (10-15x10^6^ cells in 150-300 μl) were then injected via the retro-orbital sinus. Animals were placed in a clean cage and monitored until they were fully awake and active. Three mice did not survive irradiation and BMT. Animals were given a 4 weeks of recovery prior to initiation of HFD feeding. Due to dermatitis and severe weight loss, 3 mice were euthanatized and therefore excluded from the study. Mice were randomized into two time points, yielding four groups total: recipients receiving C57BL/6 bone marrow cells and fed a HFD for 6 weeks (FWT-6, n=12) or 12 weeks (FWT-12, n=10), and recipients receiving MKP1^-/-^ bone marrow cells fed a HFD for 6 weeks (FKP-6, n=15) or 12 weeks (FKP-12, n=15).

**Plasma Cholesterol, Triglycerides, and Lipoprotein Profile**

Mice were fasted overnight prior to euthanasia and blood was collected by cardiac puncture. After 6 weeks, complete blood count analyses were run on whole blood samples to ensure reconstitution. No differences in blood cell counts were observed between groups. Blood was centrifuged and plasma collected for analysis. Plasma cholesterol and triglycerides were quantified using enzymatic assay kits per manufacturer’s protocol (Wako Chemicals USA, Inc., Richmond, VA).

Plasma was pooled from each treatment group and centrifuged to clarify the sample and 100 μl of pooled plasma was used for size exclusion chromatography. An ÄKTA FPLC and a Superose™ 6 10/300 column (GE Healthcare, Pittsburgh, PA) were used at 20-25˚C with a flow rate of 0.5 ml/min. Running buffer contained 1 mmol/L EDTA (EMD Millipore, Billerica, MA), 0.15 mol/L NaCl (Sigma-Aldrich®, St. Louis, MO), and 0.02% wt/vol NaN_3_ (Sigma-Aldrich®, St. Louis, MO), with the pH adjusted to 8.0. Fractions of 500 μl were collected and stored at -20˚C prior to total cholesterol analysis.

**In Vivo Macrophage Recruitment Assay**

Each mouse received two matrigel plugs three days prior to euthanasia as described previously [^1^](#_ENREF_1). Briefly, subcutaneous injections of Matrigel were made on the right and left flank of each mouse, one plug containing MCP-1 (500 ng/ml) and one plug containing vehicle. After euthanasia, plugs were surgically removed and digested with dispase (BD Biosciences, Franklin Lakes, NJ). Cells were stained with calcein/AM (1:1;Invitrogen, Grand Island, NY) and counted using an automated fluorescent cell counter (Nexcelcom Bios, Lawrence, MA).

**Histological and Immunohistochemical Analyses of Heart, Aorta, and Aortic Root**

After peritoneal lavage, the chest cavity was opened and the heart and aorta were perfused via the left ventricle with 10 ml PBS followed by 10 ml of ice-cold 4% paraformaldehyde (PFA) in PBS. With the heart intact, the entire aorta (extending 5 mm after bifurcation of the iliac artery, including the subclavian artery, right, and left carotid arteries) was dissected free of fat and removed.

Hearts were separated from the aorta and embedded in Tissue-Tek® Optimal Cutting Temperature compound (OCT; SAKURA Finetek USA, Inc., Torrance, CA) in a plastic cryosection mold. Hearts were situated with the axis of the aorta perpendicular to the base of the mold and were rapidly snap frozen in isopentane with dry-ice and then stored at - 80°C until further processing.

The aortas (proximal ascending aorta to the bifurcation) were fixed in 4% PFA for 48 h prior to staining for *en face* analysis. To determine the extent of the atherosclerosis, aortas were stained with Oil Red O (ORO; Sigma-Aldrich, St. Louis, MO) and digitally imaged with a camera connected to a dissecting microscope (Leica). Images were taken to include the entire aorta. Next, the aortas were opened longitudinally and digitally imaged at fixed magnification. Total aortic area and lesion area were calculated using Image Pro Plus (version 6.3; Media Cybernetics, Warrendale, PA) and ImageJ (version 1.47; NIH, Bethesda, Maryland). Lesion area is expressed as a percent of total aortic area ± S.E.

Serial sections were cut starting at the level of the aortic sinus to a depth of 420 μm. For each mouse, 6 sections (7 μm thick; separated by 80 μm) were examined. For assessment of lesion morphology one in every four slides was fixed in formalin for 10 min at room temperature and stained with hematoxylin and eosin (H&E). Using light microscopy, the number of lesions, lesion area, and necrotic area were quantified.

To further characterize lesions, neutral and intracellular lipid content were quantified by staining sections of heart tissue with ORO. Tissues were counterstained with H&E and imaged using a dissecting microscope (Leica) with an attached digital camera. Atherosclerotic lesions were analyzed using Image Pro Plus (version 6.3; Media Cybernetics, Bethesda, MD). Aortic lesion area was quantified by averaging the total lesion area of all 6 sections. Lesion area is expressed as millimeters squared. Necrotic areas were defined as those regions of the lesions that lacked nuclei and cytoplasm and are expressed as millimeters squared.

The remaining slides were air dried overnight and fixed in fresh acetone for 10 s at room temperature. Acetone-fixed sections were wrapped in aluminum foil and stored at -80ºC or processed immediately for immunohistochemistry. Sections of aortic root were blocked with 10% normal Goat serum and incubated overnight at 4°C with rat anti-mouse CD68:biotin (1:200; Bio-Rad Laboratories, Inc., Raleigh, NC) primary antibody. Tissues were also stained with antibodies to detect Signaling adaptor p62 (1:200; SQSTM1, Pierce, Rockford, IL), LC3B (1:200; Pierce, Rockford, IL), and the phosphorylation status of signal transducer and activator of transcription 1 (STAT1) at Ser 727 [Phospho-Stat1 (Ser727); 1:100; Cells Signaling Technology, Boston, MA)]. Tissues were then stained with Cy3-conjugated AffinitPure Goat Anti-Rabbit IgG and Cy2-conjugated streptavidin secondary antibody (1:500; Jackson ImmunoResearch Laboratories, West Grove, PA). In addition to visualize nuclei, tissues were stained with trihydrochloride, trihydrate (1:1000; Molecular Probes ®, Life Technologies, Grand Island, NY). Images were captured using a fluorescent microscope (Leica: DM1000) and an Olympus Camera. Non-specific staining was assessed by omitting the primary antibody. Sections were analyzed under a fluorescent microscope.

Selected sections of the aortic root were used for the detection of apoptotic and necrotic cells in atherosclerotic lesions. Tissues were analyzed by dUTP terminal nick end-labeling (TUNEL assay; Roche Diagnostics, Indianapolis, IN) according to the manufacturer’s instruction, and nuclei were counterstained with trihydrochloride, trihydate (Hoechst; 1:1000; Molecular Probes ®, Life Technologies, Grand Island, NY). Fluorescent images were captured at fixed magnification and analyzed using Photoshop software (Adobe Systems, San Jose, CA). Necrotic areas were identified by areas free of H&E staining and are reported as number of apoptotic cells versus the lesion area.

**Cell Culture**

Resident peritoneal macrophages were collected from C57BL/6 and MKP1^-/-^ mice by lavage with 10 ml ice-cold medium referred to as RPMI complete from here on [1640 media containing 5 mmol/L glucose and 1 mmol/L glutamine supplemented with 1% v/v non-essential amino acids (Gibco®, Life Technologies, Grand Island, NY), 2 mmol/L L-alanyl-glutamine (GLUTAMAX™1; Gibco®, Life Technologies, Grand Island, NY), 1 mmol/L sodium pyruvate (Cellgro®, Corning, Manassas, VA), p/s (47 U/ml and 47 ug/ml respectively), 18.6 mmol/L HEPES (Gibco®, Life Technologies, Grand Island, NY)] with 2% FBS. Peritoneal cavities were lavaged twice, with a total of 20 ml, in order to maximize recovery. Cells were centrifuged and resuspended in RPMI complete containing 2% FBS at a concentration of 1x10^6^ cells/ml. Then, the cells were plated and incubated at 37˚C, 5% CO_2_, and 95% humidity for 3 h. Nonadherent cells were removed by washing three times with the medium. Adherent macrophages were cultured in RPMI complete supplemented with 10% fetal bovine serum. Bone marrow-derived macrophages (BMDMs) were generated from C57BL/6 mice. Fresh bone marrow cells were cultured for 7 days in RPMI complete medium supplemented with 10% FBS and 50 ng/ml M-CSF. Metabolic stress was induced by incubating macrophages for 24 h in RPMI complete containing 10% FBS supplemented with 100 μg/ml LDL and 20 mmol/L D-glucose (LDL+HG). M1 polarization was induced with IFN-γ plus TNF-α (PeproTech; 50 and 10 ng/ml, respectively) [^2^](#_ENREF_2). Alternative (M2) polarization was induced by stimulating cells for 24 h with IL-4 (PeproTech; 10 ng/ml) [^2^](#_ENREF_2). Macrophages that were grown in the absence of the above cytokines were considered nonpolarized or M0. Cell viability was assessed by trypan blue dye exclusion tests. The activation of caspase-3/caspase-7 was quantified in live cells using Magic Red caspase-3/caspase-7 (AbD Serotec, Raleigh, NC) according to manufacture recommendation.

**Lentiviral Transduction of Macrophages**

pHIV-Flag-MKP-1-IRES-EGFP lentiviral plasmid was constructed by transferring the Flag-MKP-1 gene from pcDNA3.1-Flag-MKP-1 into pHIV-EGFP lentiviral plasmid (Addgene Plasmid # 21373, Cambridge, MA). All lentiviral supernatants were prepared by cotransfection of HEK-293T cells with one of the vector transfer constructs, murine ecotropic envelope vector (pCAG-Eco, Addgene Plasmid # 35617), and the packaging vectors pMDLg/pRRE and RSV-Rev. The culture medium was replaced 12 h after transfection and viral supernatants were collected 48 h post-transfection. The viral supernatants were cleared by low-speed centrifugation, filtered through a 0.45 μm syringe filter and concentrated 100-fold using a Lenti-X™ Concentrator (Cat# 631231, Clontech, Mountain View, CA). Virus titers were determined by p24 ELISA following the manufacturer's instructions (Cat# 632200, Clontech). Viral pellets were resuspended in Opti-MEM (Cat# 31985062, Invitrogen) and stored at -80°C. For gene transduction, bone marrow-derived macrophages were used on day 5 of differentiation. Two million cells were plated in 12-well plates, and viruses (MOI = 20) were added to each well in the presence of 6 µg/ml of DEAE-dextran sulfate (Sigma-Aldrich, St. Louis, MO). Cells were then incubated overnight with the vector virus, and residual vector viruses were removed by washing the wells and RPMI complete with 10% FBS was then added, and the plate was returned to the 37 °C incubator.

**Western Blot Analysis**

Cells were washed with ice-cold PBS and lysed on ice in RIPA lysis buffer (50 mmol/L Tris-HCl (pH 7.5), 150 mmol/L NaCl, 1% Nonidet P-40, 0.1% SDS, 0.5% sodium deoxycholate) supplemented with protease and phosphatase inhibitors. Aliquots with equal amounts of protein were loaded and separated on a SDS-PAGE gel. Proteins were transferred to polyvinylidene difluoride membranes (EMD Millipore, Billerica, MA) and probed using specific antibodies as indicated. Bands were detected by chemiluminescence on a KODAK Image Station 4000MM. To control for sample loading, blots were subsequently stripped and re-probed for total STAT1 or actin.

**Gene Expression Profiling**

Total RNA was isolated from cells using PureLink RNA Mini Kit (Ambion, Grand Island, NY). Reverse transcription was performed using a QuantiTect Reverse Transcription Kit (Qiagen, Valencia, CA), according to the manufacturer’s instructions. Each cDNA sample was then separated into 48 separate reactions for qPCR analysis using the BioMark 48X48 dynamic array nanofluidic chip (Fluidigm Inc., USA) according to manufacturer’s instruction. The 40 individual Taqman primer-probe mixtures (Applied Biosystems) specific for individual transcripts of interest are listed in Table S1. Amplification data were analyzed using SDS2.4 software (Applied Biosystems, Grand Island, NY), and gene expression levels were normalized to *Hprt* as the housekeeping gene.

**Statistics**

Data were analyzed using ANOVA (Sigma Stat 12.0). Data were tested for use of parametric or nonparametric post hoc analysis, and multiple comparisons were performed by using the Least Significant Difference method. All data are presented as mean ± SE of at least 3 independent experiments. Results were considered statistically significant at the *P<*0.05 level.

**SUPPLEMENTARY REFERENCES**

1. Ullevig S, Zhao Q, Lee CF, Seok Kim H, Zamora D and Asmis R. NADPH oxidase 4 mediates monocyte priming and accelerated chemotaxis induced by metabolic stress. *Arterioscler Thromb Vasc Biol*. 2012;32:415-26.

2. Tavakoli S, Zamora D, Ullevig S and Asmis R. Bioenergetic profiles diverge during macrophage polarization: implications for the interpretation of ^18^F-FDG PET imaging of atherosclerosis. *J Nucl Med*. 2013;54:1661-7.
